# An Integrated Approach to Control Soil-Transmitted Helminthiasis, Schistosomiasis, Intestinal Protozoa Infection, and Diarrhea: Protocol for a Cluster Randomized Trial

**DOI:** 10.2196/resprot.9166

**Published:** 2018-06-12

**Authors:** Giovanna Raso, Clémence Essé, Kouassi Dongo, Mamadou Ouattara, Fabien Zouzou, Eveline Hürlimann, Veronique A Koffi, Gaoussou Coulibaly, Virginie Mahan, Richard B Yapi, Siaka Koné, Jean Tenena Coulibaly, Aboulaye Meïté, Marie-Claire Guéhi-Kabran, Bassirou Bonfoh, Eliézer Kouakou N'Goran, Jürg Utzinger

**Affiliations:** ^1^ Swiss Tropical and Public Health Institute Basel Switzerland; ^2^ University of Basel Basel Switzerland; ^3^ Centre Suisse de Recherches Scientifiques en Côte d'Ivoire Abidjan Côte d'Ivoire; ^4^ Unité de Formation et de Recherche Sciences de l'Homme et de la Société Université Félix Houphouët-Boigny Abidjan Côte d'Ivoire; ^5^ Unité de Formation et de Recherche Sciences de Terre et des Ressources Minières Université Félix Houphouët-Boigny Abidjan Côte d'Ivoire; ^6^ Unité de Formation et de Recherche Biosciences Université Félix Houphouët-Boigny Abidjan Côte d'Ivoire; ^7^ FAIRMED Bern Switzerland; ^8^ UNICEF Côte d'Ivoire Abidjan Côte d'Ivoire; ^9^ Programme National de Lutte contre les Maladies Tropicales Négligées à Chimiothérapie Préventive Ministère de la Santé et de l'Hygiène Publique Abidjan Côte d'Ivoire; ^10^ Direction de l'Assainissement et du Drainage Ministère de l'Urbanisme Abidjan Côte d'Ivoire

**Keywords:** community-led total sanitation, Côte d’Ivoire, diarrhea, health education, integrated control, intestinal protozoa, preventive chemotherapy, schistosomiasis, soil-transmitted helminthiasis

## Abstract

**Background:**

The global strategy to control helminthiases (schistosomiasis and soil-transmitted helminthiasis) emphasizes preventive chemotherapy. However, in the absence of access to clean water, improved sanitation, and adequate hygiene, reinfection after treatment can occur rapidly. Integrated approaches might be necessary to sustain the benefits of preventive chemotherapy and make progress toward interruption of helminthiases transmission.

**Objective:**

The aim of this study was to assess and quantify the effect of an integrated control package that consists of preventive chemotherapy, community-led total sanitation, and health education on soil-transmitted helminthiasis, schistosomiasis, intestinal protozoa infection, and diarrhea in rural Côte d’Ivoire.

**Methods:**

In a first step, a community health education program was developed that includes an animated cartoon to promote improved hygiene and health targeting school-aged children, coupled with a health education theater for the entire community. In a second step, a cluster randomized trial was implemented in 56 communities of south-central Côte d’Ivoire with 4 intervention arms: (1) preventive chemotherapy; (2) preventive chemotherapy plus community-led total sanitation; (3) preventive chemotherapy plus health education; and (4) all 3 interventions combined. Before implementation of the aforementioned interventions, a baseline parasitologic, anthropometric, and hygiene-related knowledge, attitudes, practices, and beliefs survey was conducted. These surveys were repeated 18 and 39 months after the baseline cross-sectional survey to determine the effect of different interventions on helminth and intestinal protozoa infection, nutritional indicators, and knowledge, attitudes, practices, and beliefs. Monitoring of diarrhea was done over a 24-month period at 2-week intervals, starting right after the baseline survey.

**Results:**

Key results from this cluster randomized trial will shed light on the effect of integrated approaches consisting of preventive chemotherapy, community-led total sanitation, and health education against infections with soil-transmitted helminths, schistosomes, an intestinal protozoa and prevention of diarrhea in a rural part of Côte d’Ivoire.

**Conclusions:**

The research provided new insights into the acceptability, strengths, and limitations of an integrated community-based control package targeting helminthiases, intestinal protozoa infections, and diarrhea in rural communities of Côte d’Ivoire. In the longer term, the study will allow determining the effect of the integrated control approach on infection patterns with parasitic worms and intestinal protozoa, diarrheal incidence, anthropometric measures, and hygiene-related knowledge, attitudes, practices, and beliefs.

**Trial Registration:**

International Standard Randomized Controlled Trial Number (ISRCTN): 53102033; http://www.isrctn.com/ISRCTN53102033 (Archived by WebCite at http://www.webcitation.org/6wpnXEiHo)

**Registered Report Identifier:**

RR1-10.2196/9166

## Introduction

The global strategy to control helminthiases (eg, schistosomiasis and soil-transmitted helminthiasis) emphasizes preventive chemotherapy, that is, the periodic administration of anthelmintic drugs to at-risk populations, most importantly school-aged children [1]. However, preventive chemotherapy does not prevent people from rapid reinfection with parasitic worms [2,3]. In view of the current discussion and efforts to shift from morbidity control to interruption of transmission of helminthiases and other neglected tropical diseases, ongoing efforts need to be intensified, along with concurrent implementation of complementary interventions [4-7]. Indeed, integrated approaches, combining preventive chemotherapy with water, sanitation, and hygiene and information, education, and communication, are necessary to sustain the gains made in the control of helminthiases and eventually break transmission [8-11].

In 2015, an estimated 2.4 billion people globally lacked access to improved sanitation, and the absolute number of people practicing open defecation in Africa had increased since 1990 [12]. There is evidence that a considerable part of the global burden of disease is attributable to unsafe sanitation, poor water quality, and inadequate hygiene behavior [13,14] and that improved sanitation and water supply are key factors for prevention, control, and elimination of helminthiases and diarrhea [11,15-18]. Yet, current control efforts do not take these aspects sufficiently into account. Combined interventions have shown around 35% reduction in the incidence of diarrheal diseases and helminthiases [15,19,20] with improved sanitation being particularly important [21]. Studies pertaining to the effect of improved sanitation combined with preventive chemotherapy suggest reductions of 75% and up to 90% for each of the 3 common soil-transmitted helminth species (*Ascaris lumbricoides*, hookworm, and *Trichuris trichiura*) [22,23]. Hence, sanitation and specific health education protect people from rapid reinfection, consolidate the gains of preventive chemotherapy, and are crucial for the sustainability of control programs [24-26].

In 2013, a project was launched in south-central Côte d’Ivoire with the aim to assess and quantify the effect of preventive chemotherapy, combined with either community-led total sanitation (CLTS), or health education, or both measures combined, on reinfection with soil-transmitted helminths, schistosomes, intestinal protozoa, and the incidence of diarrhea, using a cluster randomized design. CLTS was initially designed to reduce diarrhea incidence; through a participatory grassroots approach, it aims to achieve and sustain an open defecation-free status of the target community [27].

In a first step, we developed a community health education program (CHEP), including an animated cartoon entitled *Koko et les lunettes magiques* for school-aged children [28] and a health education theater targeting the entire community. The emphasis of these health education tools is placed on improving people’s hygiene behavior to prevent the transmission of neglected tropical diseases and diarrhea. In a second step, a cluster randomized trial was implemented in 56 communities of the Taabo, Djékanou, and Toumodi departments in south-central Côte d’Ivoire. Here, we present the study protocol with particular consideration to the cluster randomized trial, whose aim was to assess the effect of preventive chemotherapy combined with either CLTS or CHEP, or both on infections with soil-transmitted helminths, schistosomes, and intestinal protozoa.

## Methods

### Ethics Approval and Consent to Participate

Ethical clearance for the study was obtained from the Ethics Committee of Basel (EKBB; reference no. 300/13, date of approval: November 11, 2013) and from the ethics committee of the Ministry of Health and Public Hygiene in Côte d’Ivoire (reference no. 76-MSLS-CNER-dkn, date of approval: November 28, 2013). The trial is registered (ISRCTN53102033, date of approval: March 26, 2014). Written informed consent was obtained from each participant, with parents/guardians consenting on behalf of children younger than 18 years.

### Study Area and Participants

Between July 2011 and December 2012, an 18-month pilot project, entailing a baseline parasitologic and knowledge, attitudes, practices, and beliefs (KAPB) cross-sectional survey, followed by a cross-sectional follow-up survey, was carried out to study the effect of an integrated disease control package, consisting of preventive chemotherapy, CLTS, and health education against helminthiases and intestinal protozoa infections in 9 communities of the Taabo health and demographic surveillance system in south-central Côte d’Ivoire [29-32]. The results of this pilot project provided an indication that an integrated control package reduced the prevalence of helminth and intestinal protozoa infections and improved people’s hygiene knowledge and practice. In addition, the study results suggested that health education is an important complement, as it enhanced CLTS acceptance in the community [33].

Following this pilot project, a larger study was launched in 2013 to assess the effect of an integrated control package in a community cluster randomized trial with 4 intervention arms (preventive chemotherapy alone, or combined with either CLTS or CHEP, or both interventions simultaneously). This trial was implemented in 56 rural communities in 3 departments of south-central Côte d’Ivoire; namely, Taabo, Djékanou, and Toumodi. In this part of Côte d’Ivoire, people are mainly engaged in subsistence agriculture, whereas rubber, cocoa, and coffee are farmed as cash crops.

### Development and Validation of Health Education Tools

Before starting the cluster randomized trial in the 3 departments, 2 types of health education tools were developed, refined, and tested—an animated cartoon entitled *Koko et les lunettes magiques* [28] and a community-based health theater. For the development of the animated cartoon, a formative research was conducted with school-aged children to identify key messages to improve hygiene behavior and to prevent transmission of neglected tropical diseases that were subsequently included in the video. The research was done with school-enrolled and nonenrolled children in 8 localities in south-central and western Côte d’Ivoire, in the same regions where further studies would take place [28]. Hence, the 8 localities were excluded from further research. The animated cartoon was produced by an Abidjan-based cartoon company, in collaboration with the research team, and was tested for comprehension and acceptance with school children. Subsequently, the cartoon was validated, and its effect on helminth infections and KAPB was determined in an intervention study comprising 25 schools of western Côte d’Ivoire from 2014 to 2015. This intervention study confirmed that knowledge of school children was improved after screening the cartoon, and hence, the cartoon was deemed a useful tool for health education. However, no significant effects on helminth infections were observed in the short term.

**Figure 1:**
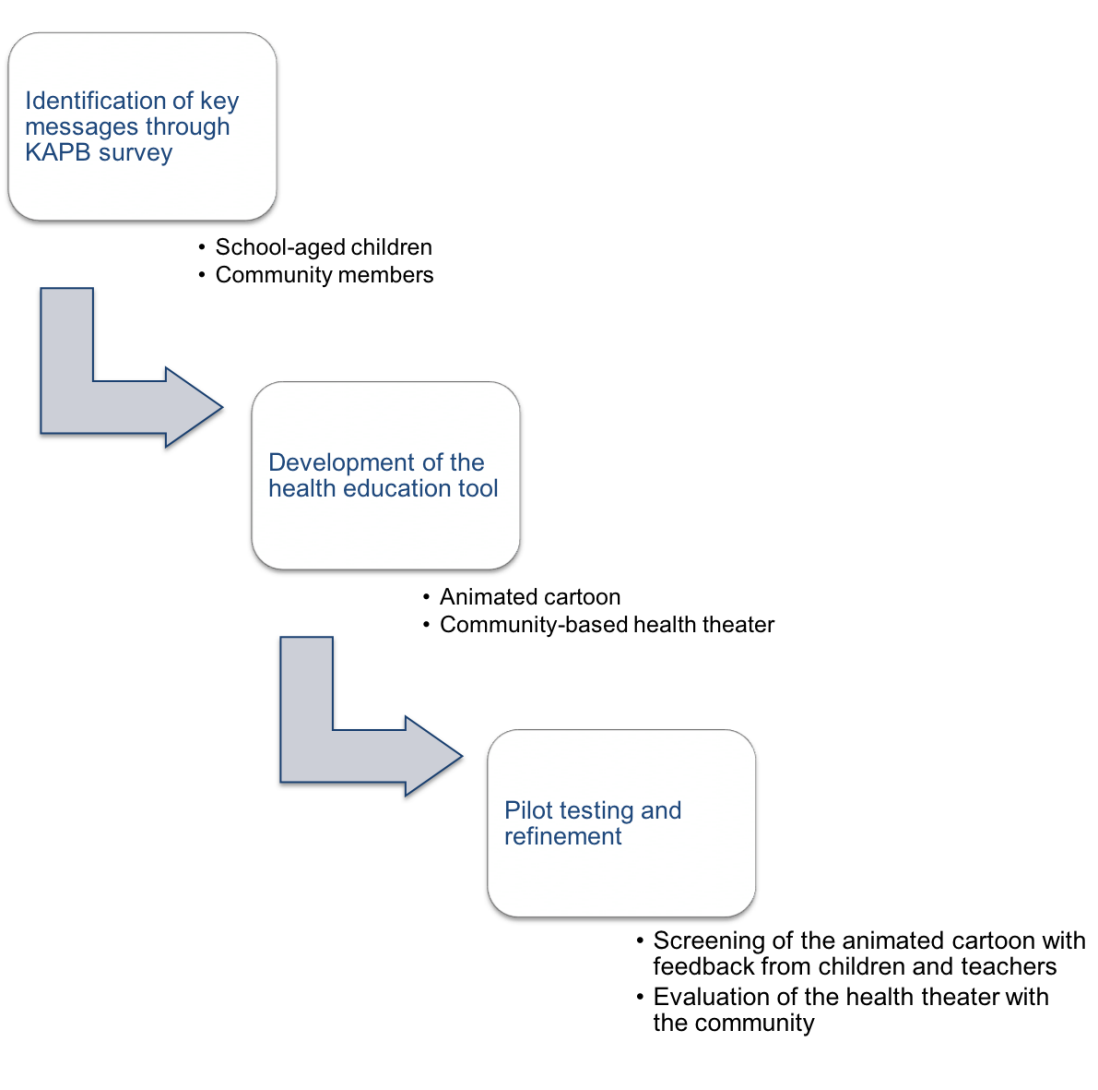
Proposed 3-step process for the development and testing of health education tools for the control of neglected tropical diseases. KAPB: knowledge, attitudes, practices, and beliefs.

As for the development of the animated cartoon, the health education theater was coupled to a KAPB survey in the community. Questionnaires and focus group discussions (FGDs) were administered to groups of women, men, young adults, and the elderly in 2 communities of the nearby Tiassalé department. In addition, direct observations were made with an emphasis on hygiene behavior and transmission of neglected tropical diseases. Community members constituted a theater group that was assisted by the research team who provided a health education session, according to KAPB survey results. The community theater members designed the sketches on their own and conveyed hygiene and health messages during their performance in front of the community. The health education theater was tested with 2 communities and then evaluated for its acceptance in the community in 2014. For this purpose, the team discussed with the community their opinion about the intervention, whether they liked it, if they thought it was helpful to improve their health knowledge, and whether they would welcome such kind of interventions. Figure 1 summarizes the 3-step methodological approach for the development of health education tools, comprising identification of key messages, development, and refinement of the tool.

### Cluster Randomized Trial Design

Once the health education tools had been developed and validated, a 4-armed cluster randomized trial was launched. The primary outcome of the trial was hookworm infection, as determined by the Kato-Katz method [34]. Secondary outcomes were other parasitic infections (ie, other soil-transmitted helminths, *Schistosoma* spp., and intestinal protozoa) and intensity of helminth infection, KAPB with regard to hygiene and intestinal parasitic infections, diarrhea incidence, and anthropometry of infants. Hookworm infection was chosen as primary outcome because of its endemicity across Côte d’Ivoire and the moderate to high prevalence in the study area [33,35].

Details of the specific outcomes are provided in the following sections. In a first step, a baseline parasitologic, KAPB, and anthropometric survey was implemented in 56 communities (14 communities per arm). Sample size calculation was done using the Web-based sample calculator for cluster randomized trials presented elsewhere [36], assuming a baseline hookworm prevalence of 30% according to previous studies in the region [33,37], a prevalence reduction of 50% after implementation of interventions [33], an intracluster correlation of .4 as we expected high correlation within the community because of the nature of community-based bottom-up interventions and mass drug administration within a community, and a dropout rate of 30% at each follow-up according to previous experience of the team, resulting in 152 individuals per cluster. In Côte d’Ivoire, the average number of people in a household is 7 (our assumption was 2 adults and 5 children). For a sample size of 152 children per community, we thus needed 30 households per community.

The communities were selected based on their population size. We intended to include communities with at least 30 households and a population size not exceeding 600 individuals because this is the optimal recommended size for implementation of the CLTS intervention [27]. Given the demographic characteristics of the study area, somewhat smaller communities (slightly less than 30 households) and villages exceeding 600 individuals were also included. The selection of up to 30 households per community was done at random, according to the World Health Organization’s (WHO) Expanded Program on Immunization method [38].

Our main target group was children aged 5 to 15 years, on whom sample size calculation was based. In addition, whenever possible, 1 infant (aged 12-24 months) and 1 adolescent or adult (aged >15 years) from each household were also selected. Although all the 3 groups underwent parasitologic examinations, only infants were subjected to anthropometric measurements. Household heads (or their spouses/partners) were administered a questionnaire for KAPB, whereas direct observations occurred in each household to check for the presence, use, and maintenance of latrines as well as potential open defecation and waste disposal sites in close proximity. The questionnaire included a section reserved for these observations that were made by the interviewer during the interview. FGDs were conducted with selected groups (adult women, adult men, school-aged children, and the elderly), and in-depth interviews were conducted with head of communities and community health workers in 24 communities. The topics discussed during the FGDs were the same as for the questionnaires so that qualitative and quantitative results complemented each other. We monitored diarrhea over a 24-month period, determining the length and frequency of each episode, using a rapid assessment questionnaire carried out once every 2 weeks. The trial communities were assigned by restricted randomization to 1 of the 4 intervention arms with 14 communities per intervention arm based on baseline soil-transmitted helminth prevalence and population size [39]. The 4 intervention arms are as follows: (1) intervention arm 1: preventive chemotherapy only; (2) intervention arm 2: preventive chemotherapy plus CLTS; (3) intervention arm 3: preventive chemotherapy plus health education; and (4) intervention arm 4: all interventions combined.

**Figure 2:**
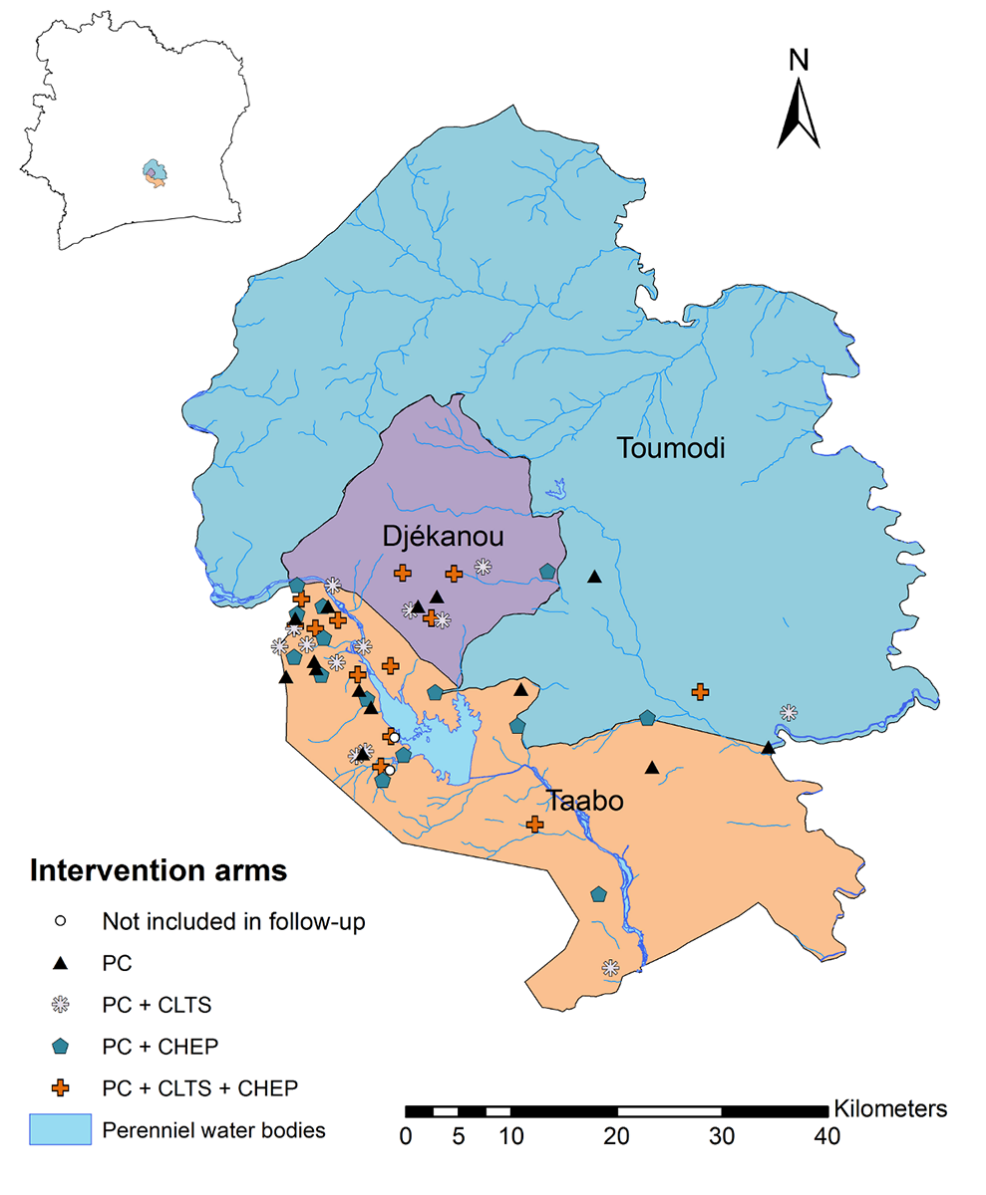
Map displaying communities included in the cluster randomized trial in 3 departments of south-central Côte d’Ivoire randomly assigned to one of 4 intervention arms. PC: preventive chemotherapy; CLTS: community-led total sanitation; CHEP: community health education program.

**Figure 3:**
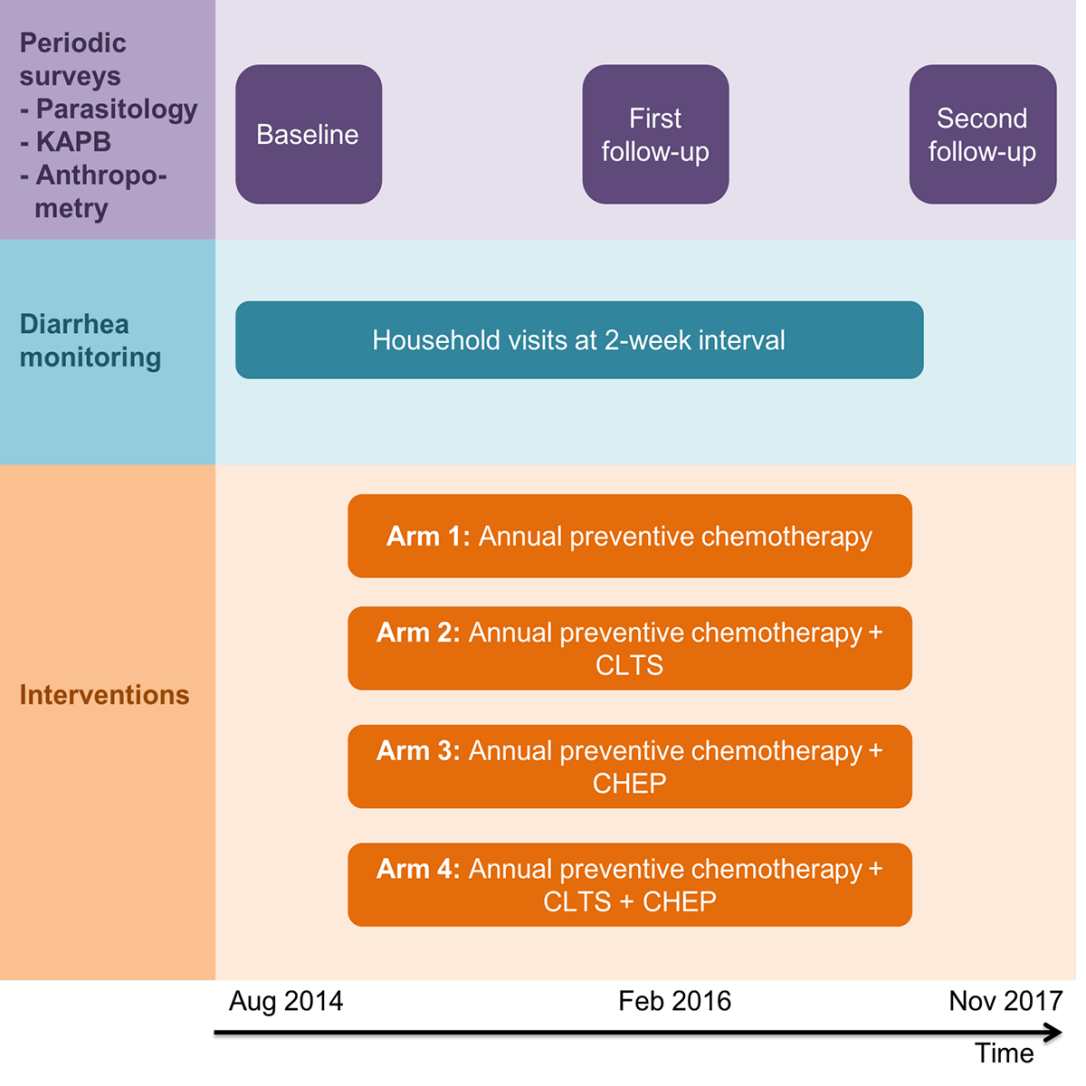
Experimental design of the cluster randomized trial. The periodic cross-sectional surveys are highlighted in purple color, diarrhea monitoring is marked in blue, and interventions are highlighted in orange. CLTS: community-led total sanitation; CHEP: community health education program; KAPB: knowledge, attitudes, practices, and beliefs.

Figure 2 shows the study area with the 56 selected rural communities, stratified by intervention arm. Interventions started right after randomization of the communities. A first follow-up parasitologic and KAPB survey was carried out 18 months after the baseline cross-sectional survey. A second follow-up survey was scheduled another 21 months later. At the end of the CLTS intervention, the communities were visited and inspected using standardized forms. Transects were done to assess whether open defecation and waste disposal spots were visible, and all households were inspected for the availability of latrines. A summary of the study design is presented in Figure 3.

### Enrollment and Written Informed Consent

For the whole study, including the parasitologic survey, preventive chemotherapy, the KAPB survey, and the interventions (CLTS and CHEP), village authorities were contacted once ethical approval had been granted. The objectives, procedures, and potential risks and benefits were explained. Subsequently, the community was informed about the aims and procedures. A patient information sheet was administered to all participants, explaining objectives, procedures, and potential risks and benefits of the study. Names and contact address of the main investigators were readily provided on this information sheet so that investigators could be contacted anytime if need be. For illiterate participants, the information sheet was read aloud, and, if necessary, an oral translation of the information into a local language was provided in the presence of a team member and a witness from the community. Written informed consent was obtained from each participant, with parents/guardians consenting on behalf of children (aged <18 years). It was emphasized that participation is voluntary, and hence, participants could withdraw from the study at any time without further obligation. Moreover, it was mentioned that preventive chemotherapy was provided to all people in the study area, not just those who decide to participate, free of charge through the national control program.

### Inclusion and Exclusion Criteria

All household heads or their representatives of the selected households of the 56 communities were invited to participate in the questionnaire survey, and all children aged 5 to 15 years, 1 infant (aged 12-24 months), and 1 adolescent or adult (aged >15 years) from these households were invited for parasitologic examination, unless they met any of the following exclusion criteria: (1) no written informed consent or no parental/guardian’s permission to participate; and (2) too sick to participate in the study, as determined by qualified medical personnel. All members of the intervention communities were invited to participate in the implementation of CLTS and the CHEP sessions.

### Cross-Sectional Surveys

Four teams were formed, each consisting of 1 driver, 2 laboratory technicians, 2 laboratory assistants, 3 field enumerators, 1 parasitologist/epidemiologist (team supervisor), and 1 social scientist. Each team was responsible for carrying out the cross-sectional parasitologic, anthropometric, and KAPB survey in their designated communities. The teams were based in 2 central laboratories of the study area, which are in close proximity to the survey locations. Moreover, 1 to 2 weeks before a cross-sectional survey, the study team visited the communities to announce the upcoming activities and to provide village authorities and inhabitants with exact dates and procedures of the survey.

### Parasitologic Surveys

A day before the first sampling, the study team conducting the survey visited the selected households and distributed empty plastic containers for stool and urine collection. The team revisited the households to collect the samples early in the morning of the next day [40]. Stool and urine samples were transferred to laboratories at the general hospitals of Taabo and Djékanou, the community health center at Kpouèbo, or a mobile field laboratory set up at the dispensary of Léléblé.

Participants’ infection status with helminths (*A. lumbricoides*, hookworm, *Schistosoma mansoni*, and *T. trichiura*), pathogenic intestinal protozoa (*Giardia intestinalis*, *Entamoeba histolytica/E. dispar*), as determined in stool samples, and *S. haematobium,* determined in urine samples, were recorded. From each stool sample, duplicate Kato-Katz thick smears were prepared, using a standard template holding 41.7 mg of feces [34]. The slides were allowed to clear for 30 to 45 min before examining under a microscope by experienced laboratory technicians. Helminth eggs were counted and recorded for each species separately. For quality control, approximately 10% of the slides, selected at random, were reexamined by a senior laboratory technician [41]. Urine samples were examined for microhematuria using reagent strips (Hemastix; Siemens Healthcare Diagnostics GmbH, Eschborn, Germany). A subsample of 10% of urine specimens was subjected to a filtration method for evaluation of the reagent strip results. Ten milliliters of vigorously shaken urine were pressed through a membrane (diameter: 13 mm; pore size: 30 µm; Sefar AG, Heiden, Switzerland) and the membrane placed on a microscope slide. A drop of Lugol’s iodine was added on the slide, and the number of *S. haematobium* eggs was counted under a microscope by experienced laboratory technicians [42].

In addition, 1 to 2 g of stool from each specimen was transferred into small tubes, filled with 10 mL of sodium acetate-acetic acid-formalin (SAF) for subsequent diagnosis of intestinal protozoa. In short, the SAF-fixed stool samples were forwarded to a laboratory at the Université Félix Houphouët-Boigny in Abidjan and subjected to an ether-concentration method and examined under a microscope by experienced laboratory technicians. We adhered to a standard protocol [43].

### Anthropometric Measurements

In intervention arms 1 and 4, infants aged 12 to 24 months were assessed for standard anthropometric measures, including weight (to the nearest 0.1 kg; mothers holding their infant were weighed with a portable scale, and then the weight of the mother was subtracted to obtain the weight of the infant) and height (measured to the nearest cm using a portable centimeter scale). Nutritional status of children at baseline and follow-up was evaluated using the following indicators: underweight (weight for age), stunting (height for age), and wasting (weight for height).

### Knowledge, Attitudes, Practices, and Beliefs Surveys

People’s KAPB were assessed, using a combination of direct observations and interviews (questionnaire surveys, in-depth interviews, and FGDs). All the components of the KAPB study were conducted in parallel to the baseline and follow-up parasitologic and anthropometric surveys.

The main topics that were investigated in the KAPB survey pertained to perceived needs of sanitation facilities, common defecation practices, availability and use of latrines, associations of defecation and hygiene behavior (eg, washing hands), general knowledge of health risks associated with (open) defecation, signs and symptoms of parasite infections, and how such infections can be prevented and treated [24]. Direct observations and questionnaires were addressed to household heads or their representatives at the unit of the household in the 56 communities. Questionnaires were designed in a semistructured manner with mainly closed but also a few open-ended questions to gather quantitative and qualitative data for the analyses. All interviews were conducted by trained field enumerators in French or local languages. Before the start of the survey, the questionnaire and the direct observation checklist were pretested in neighboring communities that were not part of the study, as done in previous research [44,45].

FGDs were conducted with different groups; namely (1) adult women; (2) adult men; (3) school-aged children; and (4) the elderly. FGDs were conducted in 8 villages; 2 villages per intervention arm. In each FGD, 8 to 10 individuals were invited to participate [46]. FGDs were tape-recorded for subsequent transcription and analysis. In-depth interviews were conducted with community health workers and traditional healers in the same 8 villages.

### Diarrhea Monitoring

We monitored the incidence of diarrhea (duration and severity) over a 24-month period. Every second week, a short questionnaire was administered by community health workers to all members of the 30 selected households per community, starting right after the baseline cross-sectional survey. For the youngest children who were not able to answer the questionnaire, their mothers/caregivers were interviewed.

### Implementation of Interventions

The interventions were implemented after the baseline cross-sectional survey. The CLTS intervention was started in the communities of intervention arms 2 and 4. Only after these communities had commenced building latrines, the CHEP was launched in the communities of intervention arms 3 and 4 to avoid interference with the methodological approach of the CLTS intervention (see section Community-Led Total Sanitation) in arm 4. Preventive chemotherapy was done according to ongoing activities of the national helminthiasis control program of the Ministry of Health in Côte d’Ivoire. These activities consist of community-based yearly mass administration of ivermectin and albendazole (against lymphatic filariasis) and yearly administration of praziquantel and albendazole (against schistosomiasis and soil-transmitted helminthiasis) to at-risk groups, adhering to WHO guidelines [47].

### Preventive Chemotherapy

After the baseline cross-sectional survey, in October 2014, all participants found positive for *S. mansoni,* or *S. haematobium*, or both, received a single 40 mg/kg oral dose of praziquantel using a dose pole for individuals aged 4 years and older, whereas albendazole (single 400 mg dose for participants aged >2 years and 200 mg for 1- to 2-years-old children) was administered against soil-transmitted helminths [48]. Thereafter, annual preventive chemotherapy was administered in the frame of the on-going helminthiasis control activities by the Ministry of Health in Côte d’Ivoire. Annual preventive chemotherapy against lymphatic filariasis was done between May and June and against schistosomiasis between October and November. Participants with persistent diarrhea identified during the diarrhea monitoring received oral rehydration solutions and, if needed, were referred to nearby health facilities.

### Community-Led Total Sanitation

The CLTS approach is based on participatory rural appraisal that emphasizes that the learning effect is considerably higher if knowledge is acquired through self-experience and self-reflexion. It facilitates critical analysis by the community of their own sanitation profile, their practices of defecation, and the consequences, leading to collective action to become open defecation-free [49,50]. The approach thus focuses on the whole community and their cooperation and interactions because the community only profits when every single community member cooperates and takes action [51]. CLTS is a grassroots, community-based, and community-led strategy that triggers community empowerment via feelings of shame and disgust induced through observation of the defecation situation in a specific setting and its environment, which is often missed by health education [52,53].

Before the intervention, CLTS facilitators were trained and instructed during a 1-week workshop by certified national CLTS facilitators from the Ministry of Sanitation, UNICEF, and a Swiss-based nongovernmental organization (FAIRMED). Communities were contacted, and a first community meeting was organized. During this meeting, the ignition process of CLTS was started, which could include the following components according to Kar and Chambers [27]: (1) transect walk (“walk of shame”) through the open defecation areas and water points; (2) defecation map, mapping of defecation areas and defecation mobility; (3) identifying the dirtiest neighborhoods; (4) calculation of feces amount and medical expenses; and (5) triggering disgust pathways of fecal contamination (glass of water, feces to food exercise, flow diagrams of fecal-oral routes, etc). These components were used to initiate the ignition moment (we are eating each other’s feces!). When talking about feces, the term “caca” was employed, as “caca” had been identified as the most suited and culturally accepted term by CLTS facilitators in Côte d’Ivoire. Subsequently, 2 more steps were pursued that include the identification of natural leaders and the monitoring and sustaining of open defecation-free status or the process toward an open defecation-free community. Although CLTS encourages the communities to build basic sanitary facilities (ie, latrines), to change one’s hygiene behavior, and to alter people’s waste disposal practices, it does not impose standard designs and does not provide subsidies. Hence, for toilet construction, most communities used readily available local material, so that toilets were more affordable and accessible for rural communities. When a given community decided to go toward open defecation-free status, an implementation plan of community-based basic sanitation and hygiene services was elaborated. This community was frequently visited by the community development agents to ensure that the implementation of the different actions was done according to protocol.

### Community Health Education Program

Once communities were well advanced or staggered with the construction of latrines, they were visited by the research team, and a first health education session was offered for the entire community, based on results from the preceding FGDs that facilitated the social science team to identify knowledge gaps and related key health messages. In each community, interested community members (up to 10) were identified (mostly by the village chief and other village authorities) and invited to form a community health theater group. In a further visit, the community theater group received an additional health education session by the team and was coached to develop its own sketch to deliver hygiene and health messages in front of the community. In a final visit, the theater group presented the sketch to the community, and during this visit, the animated cartoon was screen played to children, although adults were also invited to watch. After the theater and screening of the cartoon, people were grouped into adult women, adult men, school-aged children, and the elderly, and discussions about health and hygiene topics were pursued to determine their understanding.

### Statistical Analysis

Data collected from the parasitologic and anthropometric surveys and the monitoring of diarrheal episodes were double entered and cross-checked in EpiInfo version 3.5.3 (Centers for Disease Control and Prevention; Atlanta, GA, USA). Household questionnaire data and direct observations were entered on tablets, using open data kit, and uploaded on a server hosted at the Swiss Tropical and Public Health Institute (Swiss TPH; Basel, Switzerland). Statistical analyses were done on STATA, version 14 (Stata Corp; College Station, TX, USA).

To receive a standard infection intensity measure of eggs per 1 g (EPG) of stool, helminth species–specific egg counts from the individual Kato-Katz thick smears were multiplied by a factor of 24. For each individual, the arithmetic mean egg count was estimated. The geometric mean of the helminth infection at the population level was calculated from the arithmetic means of the individual infection intensities. To assess the effect of the interventions, helminth egg count reductions were determined as *1 − (geometric mean EPG after 1 year at follow-up/geometric mean EPG at baseline) multiplied by a factor 100* and compared between intervention groups.

The nutritional status for children aged <5 years was determined using available macros for STATA version 10.1 with child growth standards and references published by WHO [54] and means between intervention groups, compared between baseline and follow-up. The same approach was used for diarrheal episodes, whereas the prevalence is being defined as the percentage of days with diarrhea, calculated as the number of days with diarrhea divided by the total number of days of observation. Incidence is defined as the number of new episodes divided by the number of days at-risk, which is defined as the number of days of observation minus the number of days with diarrhea self-diagnosis, allowing for 2 illness-free days between episodes [55].

Random effect logistic regression models were used to assess the effect of interventions on infection, anthropometric, diarrhea, and KAPB outcomes, using a factorial design. Qualitative data gathered from the FGDs were transcribed and processed in MaxQDA 10/Atlas version 1 (VERBI Software Consult; Berlin, Germany). The coded data were analyzed for the frequency at which coded information and content categories occur. The most frequently occurring topics concerning the study population’s KAPB were analyzed for change after the implementation of CLTS and/or CHEP.

### Dissemination of Key Findings

Progress and key results of this cluster randomized trial were communicated at annual workshops with key decision makers and other stakeholders, including community members.

## Results

The project was funded in May 2013 and enrollment was completed in September 2014. Baseline, follow-up I, and follow-up II surveys were completed in September 2014, February 2016, and November 2017, respectively. Data analysis is currently under way, and the first results are expected to be submitted for publication in 2018. The findings will be published in peer-reviewed literature and presented at national and international conferences.

## Discussion

Neglected tropical diseases, including soil-transmitted helminthiasis, schistosomiasis, giardiasis, and amoebiasis, are important public health issues in Côte d’Ivoire and elsewhere in low- and middle-income countries [14,56-58]. Indeed, a recent national school-based survey in 94 schools across Côte d’Ivoire revealed that 26% of children aged 5 to 15 years had a helminth infection [59]. Giardiasis and amoebiasis were reported from community- and school-based surveys in different parts of Côte d’Ivoire [30,60,61]. The aim of this project was to assess the effect of an integrated control package, consisting of preventive chemotherapy with either CLTS or CHEP, or all measures combined in 56 small rural communities of south-central Côte d’Ivoire, using a cluster randomized trial. The goal was to generate new evidence to determine whether an integrated control package, including community-based approaches, is useful for the control of helminth and intestinal protozoa infections and thus to assist decision making in translating global policy into local practice.

A previous pilot study in Côte d’Ivoire revealed that CLTS coupled with health education and preventive chemotherapy has the potential to decrease the incidence of helminth and intestinal protozoa infection, although heterogeneity from one community to another rendered interpretation of the results somewhat difficult [33]. Notwithstanding, a recent cluster randomized study in Mali found no effect of CLTS on diarrhea 18 months after implementation of the intervention, but a significant beneficial effect on children’s anthropometric measures, as children from the intervention group were significantly less stunted [62]. Of note, the authors used a cross-sectional design for assessing diarrheal incidence at 2 time points (baseline and end line). In this protocol, diarrhea was monitored longitudinally over a 24-month period with 2-week intervals, which should capture subtle fluctuations. Two recent cluster randomized trials from India showed no effect on diarrhea, soil-transmitted helminths, and child malnutrition and highlighted the difficulty to achieve high coverage of latrine use at a large scale to demonstrate expected health outcomes [63,64].

There is considerable interest in the scientific community and among disease control program managers to bring integrated approaches into action, although the challenges of scaling up such integrated, intersectoral, multidisease control approaches are recognized [65,66]. CLTS holds promise to decrease diarrhea, helminthiases, and intestinal protozoa infections, yet, limitations with regard to achieving open defecation-free status and sustainability exist. Our previous work in Côte d’Ivoire has provided evidence that health education interventions can improve adherence of communities to CLTS and thus increase the success rate of such an intervention [33]. A further limitation of our study is that the distance between communities was sometimes relatively small, and contamination cannot be completely excluded. Although, it has to be emphasized that in this particular study area, the difficult physical accessibility to communities can limit contamination even if communities are relatively close. Furthermore, although during the CLTS and CHEP interventions, it was emphasized that a hand washing facility needs to be provided next to the latrines (eg, bucket with water and soap) and before eating, hands need to be washed with soap, no specific water access intervention was included in the study, which might have an impact on infection outcomes. Finally, sample size was limited by the size of the communities because of the methodological approach of CLTS. Indeed, communities up to a maximum size of 500 to 600 members are more likely to adhere to CLTS compared with larger communities.

This study and experiences gained elsewhere [67] will shed new light on the effect of integrated approaches on different outcomes, including parasitic infections (soil-transmitted helminths, schistosomes, and intestinal protozoa), incidence of diarrhea, anthropometric measures, and KAPB of populations. Furthermore, this line of scientific inquiry will enhance our knowledge of community acceptance regarding integrated control approaches, including strengths and limitations, and provide important information for existing sanitation and health programs in Côte d’Ivoire and elsewhere.

## Abbreviations

CHEP: community health education program
CLTS: community-led total sanitation
EPG: eggs per 1 g
FGDs: focus group discussions
KAPB: knowledge, attitudes, practices, and beliefs
SAF: sodium acetate-acetic acid-formalin
WHO: World Health Organization

## References

[1] World Health Organization (2006). Preventive chemotherapy in human helminthiasis; coordinated use of anthelminthic drugs in control interventions: a manual for health professionals and programme managers.

[2] Quinnell RJ, Slater AF, Tighe P, Walsh EA, Keymer AE, Pritchard DI (1993). Reinfection with hookworm after chemotherapy in Papua New Guinea. Parasitology.

[3] Jia TW, Melville S, Utzinger J, King CH, Zhou XN (2012). Soil-transmitted helminth reinfection after drug treatment: a systematic review and meta-analysis. PLoS Negl Trop Dis.

[4] Knopp S, Stothard JR, Rollinson D, Mohammed KA, Khamis IS, Marti H, Utzinger J (2013). From morbidity control to transmission control: time to change tactics against helminths on Unguja Island, Zanzibar. Acta Trop.

[5] Rollinson D, Knopp S, Levitz S, Stothard JR, Tchuem Tchuenté LA, Garba A, Mohammed KA, Schur N, Person B, Colley DG, Utzinger J (2013). Time to set the agenda for schistosomiasis elimination. Acta Trop.

[6] Ross AG, Chau TN, Inobaya MT, Olveda RM, Li Y, Harn DA (2017). A new global strategy for the elimination of schistosomiasis. Int J Infect Dis.

[7] World Health Organization (2017). Integrating Neglected Tropical Diseases in Global Health and Development.

[8] Mara D, Lane J, Scott B, Trouba D (2010). Sanitation and health. PLoS Med.

[9] Knopp S, Mohammed KA, Ali SM, Khamis IS, Ame SM, Albonico M, Gouvras A, Fenwick A, Savioli L, Colley DG, Utzinger J, Person B, Rollinson D (2012). Study and implementation of urogenital schistosomiasis elimination in Zanzibar (Unguja and Pemba islands) using an integrated multidisciplinary approach. BMC Public Health.

[10] Bieri FA, Gray DJ, Williams GM, Raso G, Li YS, Yuan L, He Y, Li RS, Guo F, Li SM, McManus DP (2013). Health-education package to prevent worm infections in Chinese schoolchildren. N Engl J Med.

[11] Strunz EC, Addiss DG, Stocks ME, Ogden S, Utzinger J, Freeman MC (2014). Water, sanitation, hygiene, and soil-transmitted helminth infection: a systematic review and meta-analysis. PLoS Med.

[12] World Health Organization, United Nations International Children's Emergency Fund (2015). Progress on Sanitation and Drinking Water: 2015 Update and MDG Assessment.

[13] Prüss-Üstün A, Bos R, Gore F, Bartram J (2008). Safe water, better health: cost, benefits and sustainability of interventions to protect and promothe health.

[14] GBD 2015 Disease and Injury Incidence and Prevalence Collaborators (2016). Global, regional, and national incidence, prevalence, and years lived with disability for 310 diseases and injuries, 1990-2015: a systematic analysis for the Global Burden of Disease Study 2015. Lancet.

[15] Esrey SA, Potash JB, Roberts L, Shiff C (1991). Effects of improved water supply and sanitation on ascariasis, diarrhoea, dracunculiasis, hookworm infection, schistosomiasis, and trachoma. Bull World Health Organ.

[16] Bartram J, Cairncross S (2010). Hygiene, sanitation, and water: forgotten foundations of health. PLoS Med.

[17] Grimes JE, Croll D, Harrison WE, Utzinger J, Freeman MC, Templeton MR (2014). The relationship between water, sanitation and schistosomiasis: a systematic review and meta-analysis. PLoS Negl Trop Dis.

[18] Freeman MC, Clasen T, Brooker SJ, Akoko DO, Rheingans R (2013). The impact of a school-based hygiene, water quality and sanitation intervention on soil-transmitted helminth reinfection: a cluster-randomized trial. Am J Trop Med Hyg.

[19] Fewtrell L, Kaufmann RB, Kay D, Enanoria W, Haller L, Colford JM (2005). Water, sanitation, and hygiene interventions to reduce diarrhoea in less developed countries: a systematic review and meta-analysis. Lancet Infect Dis.

[20] Cairncross S, Hunt C, Boisson S, Bostoen K, Curtis V, Fung IC, Schmidt WP (2010). Water, sanitation and hygiene for the prevention of diarrhoea. Int J Epidemiol.

[21] Prüss A, Kay D, Fewtrell L, Bartram J (2002). Estimating the burden of disease from water, sanitation, and hygiene at a global level. Environ Health Perspect.

[22] Arfaa F, Sahba GH, Farahmandian I, Jalali H (1977). Evaluation of the effect of different methods of control of soil-transmitted helminths in Khuzestan, southwest Iran. Am J Trop Med Hyg.

[23] Udonsi JK, Ogan VN (1993). Assessment of the effectiveness of primary health care interventions in the control of three intestinal nematode infections in rural communities. Public Health.

[24] Asaolu SO, Ofoezie IE (2003). The role of health education and sanitation in the control of helminth infections. Acta Trop.

[25] Albonico M, Montresor A, Crompton DW, Savioli L (2006). Intervention for the control of soil-transmitted helminthiasis in the community. Adv Parasitol.

[26] Acka CA, Raso G, N'Goran EK, Tschannen AB, Bogoch II, Séraphin E, Tanner M, Obrist B, Utzinger J (2010). Parasitic worms: knowledge, attitudes, and practices in western Côte d'Ivoire with implications for integrated control. PLoS Negl Trop Dis.

[27] Kar K, Chambers R (2008). Handbook on community-led total sanitation.

[28] Essé C, Koffi VA, Kouamé A, Dongo K, Yapi RB, Moro HM, Kouakou CA, Palmeirim MS, Bonfoh B, N'Goran EK, Utzinger J, Raso G (2017). “Koko et les lunettes magiques”: an educational entertainment tool to prevent parasitic worms and diarrheal diseases in Côte d'Ivoire. PLoS Negl Trop Dis.

[29] Fürst T, Silué KD, Ouattara M, N'Goran DN, Adiossan LG, N'Guessan Y, Zouzou F, Koné S, N'Goran EK, Utzinger J (2012). Schistosomiasis, soil-transmitted helminthiasis, and sociodemographic factors influence quality of life of adults in Côte d'Ivoire. PLoS Negl Trop Dis.

[30] Schmidlin T, Hürlimann E, Silué KD, Yapi RB, Houngbedji C, Kouadio BA, Acka-Douabélé CA, Kouassi D, Ouattara M, Zouzou F, Bonfoh B, N'Goran EK, Utzinger J, Raso G (2013). Effects of hygiene and defecation behavior on helminths and intestinal protozoa infections in Taabo, Côte d'Ivoire. PLoS One.

[31] Koné S, Baikoro N, N'Guessan Y, Jaeger FN, Silué KD, Fürst T, Hürlimann E, Ouattara M, Séka MY, N'Guessan NA, Esso EL, Zouzou F, Boti LI, Gonety PT, Adiossan LG, Dao D, Tschannen AB, von Stamm T, Bonfoh B, Tanner M, Utzinger J, N'Goran EK (2015). Health & demographic surveillance system profile: the Taabo health and demographic surveillance system Côte d'Ivoire. Int J Epidemiol.

[32] Koné S, Fürst T, Jaeger FN, Esso EL, Baïkoro N, Kouadio KA, Adiossan LG, Zouzou F, Boti LI, Tanner M, Utzinger J, Bonfoh B, Dao D, N'Goran EK (2015). Causes of death in the Taabo health and demographic surveillance system, Côte d'Ivoire, from 2009 to 2011. Glob Health Action.

[33] Hürlimann E, Silué KD, Zouzou F, Ouattara M, Schmidlin T, Yapi RB, Houngbedji CA, Dongo K, Kouadio BA, Koné S, Bonfoh B, N'Goran EK, Utzinger J, Acka-Douabélé CA, Raso G (2018). Effect of an integrated intervention package of preventive chemotherapy, community-led total sanitation and health education on the prevalence of helminth and intestinal protozoa infections in Côte d'Ivoire. Parasit Vectors.

[34] Katz N, Chaves A, Pellegrino J (1972). A simple device for quantitative stool thick-smear technique in schistosomiasis mansoni. Rev Inst Med Trop Sao Paulo.

[35] Yapi RB, Chammartin F, Hürlimann E, Houngbedji CA, N'Dri PB, Silué KD, Utzinger J, N'Goran EK, Vounatsou P, Raso G (2016). Bayesian risk profiling of soil-transmitted helminth infections and estimates of preventive chemotherapy for school-aged children in Côte d'Ivoire. Parasit Vectors.

[36] Campbell MK, Thomson S, Ramsay CR, MacLennan GS, Grimshaw JM (2004). Sample size calculator for cluster randomized trials. Comput Biol Med.

[37] Becker SL, Sieto B, Silué KD, Adjossan L, Koné S, Hatz C, Kern WV, N'Goran EK, Utzinger J (2011). Diagnosis, clinical features, and self-reported morbidity of
*Strongyloides stercoralis* and hookworm infection in a co-endemic setting. PLoS Negl Trop Dis.

[38] Lemeshow S, Tserkovnyi AG, Tulloch JL, Dowd JE, Lwanga SK, Keja J (1985). A computer simulation of the EPI survey strategy. Int J Epidemiol.

[39] Moulton LH (2004). Covariate-based constrained randomization of group-randomized trials. Clin Trials.

[40] Raso G, N'Goran EK, Toty A, Luginbühl A, Adjoua CA, Tian-Bi NT, Bogoch II, Vounatsou P, Tanner M, Utzinger J (2004). Efficacy and side effects of praziquantel against
*Schistosoma mansoni* in a community of western Côte d'Ivoire. Trans R Soc Trop Med Hyg.

[41] Montresor A, Crompton DWT, Bundy DAP, Hall A, Savioli L (1998). Guidelines for the evaluation of soil-transmitted helminthiasis and schistosomiasis at community level: a guide for managers of control programmes.

[42] Savioli L, Hatz C, Dixon H, Kisumku U, Mott K (1990). Control of morbidity due to
*Schistosoma haematobium* on Pemba Island: egg excretion and hematuria as indicators of infection. Am J Trop Med Hyg.

[43] Utzinger J, Botero-Kleiven S, Castelli F, Chiodini P, Edwards H, Köhler N, Gulletta M, Lebbad M, Manser M, Matthys B, N'Goran E, Tannich E, Vounatsou P, Marti H (2010). Microscopic diagnosis of sodium acetate-acetic acid-formalin-fixed stool samples for helminths and intestinal protozoa: a comparison among European reference laboratories. Clin Microbiol Infect.

[44] Utzinger J, N'Goran EK, Ossey YA, Booth M, Traoré M, Lohourignon KL, Allangba A, Ahiba LA, Tanner M, Lengeler C (2000). Rapid screening for
*Schistosoma mansoni* in western Côte d'Ivoire using a simple school questionnaire. Bull World Health Organ.

[45] Raso G, Luginbühl A, Adjoua CA, Tian-Bi NT, Silué KD, Matthys B, Vounatsou P, Wang Y, Dumas ME, Holmes E, Singer BH, Tanner M, N'goran EK, Utzinger J (2004). Multiple parasite infections and their relationship to self-reported morbidity in a community of rural Côte d'Ivoire. Int J Epidemiol.

[46] Dawson S, Manderson L, Tallo V (1993). A manual for the use of focus groups: methods for social research in disease.

[47] World Health Organization (2002). Prevention and control of schistosomiasis and soil-transmitted helminthiasis: report of a WHO expert committee.

[48] Keiser J, Utzinger J (2008). Efficacy of current drugs against soil-transmitted helminth infections: systematic review and meta-analysis. J Am Med Assoc.

[49] Chambers R (1994). The origins and practice of participatory rural appraisal. World Dev.

[50] Bongartz P, Musyoki SM, Milligan A, Ashley H (2010). Tales of shit: community-led total sanitation in Africa.

[51] Kar K, Pasteur K (2005). Subsidy or self-Respect? Community-led total sanitation. An update on recent developments.

[52] Jenkins MW, Curtis V (2005). Achieving the 'good life': why some people want latrines in rural Benin. Soc Sci Med.

[53] Jenkins MW, Scott B (2007). Behavioral indicators of household decision-making and demand for sanitation and potential gains from social marketing in Ghana. Soc Sci Med.

[54] Duggan MB (2010). Anthropometry as a tool for measuring malnutrition: impact of the new WHO growth standards and reference. Ann Trop Paediatr.

[55] Ruel MT, Rivera JA, Santizo MC, Lönnerdal B, Brown KH (1997). Impact of zinc supplementation on morbidity from diarrhea and respiratory infections among rural Guatemalan children. Pediatrics.

[56] Tchuem-Tchuenté LA, N'Goran EK (2009). Schistosomiasis and soil-transmitted helminthiasis control in Cameroon and Côte d'Ivoire: implementing control on a limited budget. Parasitology.

[57] Hotez PJ, Alvarado M, Basáñez MG, Bolliger I, Bourne R, Boussinesq M, Brooker SJ, Brown AS, Buckle G, Budke CM, Carabin H, Coffeng LE, Fèvre EM, Fürst T, Halasa YA, Jasrasaria R, Johns NE, Keiser J, King CH, Lozano R, Murdoch ME, O'Hanlon S, Pion SD, Pullan RL, Ramaiah KD, Roberts T, Shepard DS, Smith JL, Stolk WA, Undurraga EA, Utzinger J, Wang M, Murray CJ, Naghavi M (2014). The Global Burden of Disease study 2010: interpretation and implications for the neglected tropical diseases. PLoS Negl Trop Dis.

[58] Rogawski ET, Bartelt LA, Platts-Mills JA, Seidman JC, Samie A, Havt A, Babji S, Trigoso DR, Qureshi S, Shakoor S, Haque R, Mduma E, Bajracharya S, Gaffar SM, Lima AA, Kang G, Kosek MN, Ahmed T, Svensen E, Mason C, Bhutta ZA, Lang DR, Gottlieb M, Guerrant RL, Houpt ER, Bessong PO, MAL-ED Network Investigators (2017). Determinants and impact of
*Giardia* infection in the first 2 years of life in the MAL-ED birth cohort. J Pediatric Infect Dis Soc.

[59] Yapi RB, Hürlimann E, Houngbedji CA, N'Dri PB, Silué KD, Soro G, Kouamé FN, Vounatsou P, Fürst T, N'Goran EK, Utzinger J, Raso G (2014). Infection and co-infection with helminths and
*Plasmodium* among school children in Côte d'Ivoire: results from a national cross-sectional survey. PLoS Negl Trop Dis.

[60] Ouattara M, N'Guéssan NA, Yapi A, N'Goran EK (2010). Prevalence and spatial distribution of
*Entamoeba histolytica/dispar* and
*Giardia lamblia* among schoolchildren in Agboville area (Côte d'Ivoire). PLoS Negl Trop Dis.

[61] Hürlimann E, Houngbedji CA, N’Dri PB, Bänninger D, Coulibaly JT, Yap P, Silué KD, N’Goran EK, Raso G, Utzinger J (2014). Effect of deworming on school-aged children's physical fitness, cognition and clinical parameters in a malaria-helminth co-endemic area of Côte d'Ivoire. BMC Infect Dis.

[62] Pickering AJ, Djebbari H, Lopez C, Coulibaly M, Alzua ML (2015). Effect of a community-led sanitation intervention on child diarrhoea and child growth in rural Mali: a cluster-randomised controlled trial. Lancet Glob Health.

[63] Clasen T, Boisson S, Routray P, Torondel B, Bell M, Cumming O, Ensink J, Freeman M, Jenkins M, Odagiri M, Ray S, Sinha A, Suar M, Schmidt W (2014). Effectiveness of a rural sanitation programme on diarrhoea, soil-transmitted helminth infection, and child malnutrition in Odisha, India: a cluster-randomised trial. Lancet Glob Health.

[64] Patil SR, Arnold BF, Salvatore AL, Briceno B, Ganguly S, Colford JM, Gertler PJ (2014). The effect of India's total sanitation campaign on defecation behaviors and child health in rural Madhya Pradesh: a cluster randomized controlled trial. PLoS Med.

[65] Nakagawa J, Ehrenberg JP, Nealon J, Fürst T, Aratchige P, Gonzales G, Chanthavisouk C, Hernandez LM, Fengthong T, Utzinger J, Steinmann P (2015). Towards effective prevention and control of helminth neglected tropical diseases in the Western Pacific Region through multi-disease and multi-sectoral interventions. Acta Trop.

[66] Lo NC, Addiss DG, Hotez PJ, King CH, Stothard JR, Evans DS, Colley DG, Lin W, Coulibaly JT, Bustinduy AL, Raso G, Bendavid E, Bogoch II, Fenwick A, Savioli L, Molyneux D, Utzinger J, Andrews JR (2017). A call to strengthen the global strategy against schistosomiasis and soil-transmitted helminthiasis: the time is now. Lancet Infect Dis.

[67] Nery SV, McCarthy JS, Traub R, Andrews RM, Black J, Gray D, Weking E, Atkinson JA, Campbell S, Francis N, Vallely A, Williams G, Clements A (2015). A cluster-randomised controlled trial integrating a community-based water, sanitation and hygiene programme, with mass distribution of albendazole to reduce intestinal parasites in Timor-Leste: the WASH for WORMS research protocol. BMJ Open.

